# Supplementary figures and images for: Experimental inoculation of pigs with porcine parainfluenza virus 1 revealed pathological manifestations in the upper respiratory tract
Source: PLoS Pathog. 2026 Mar 30;22(3):e1013405. doi: 10.1371/journal.ppat.1013405 (PMC13056255; doi:10.1371/journal.ppat.1013405)

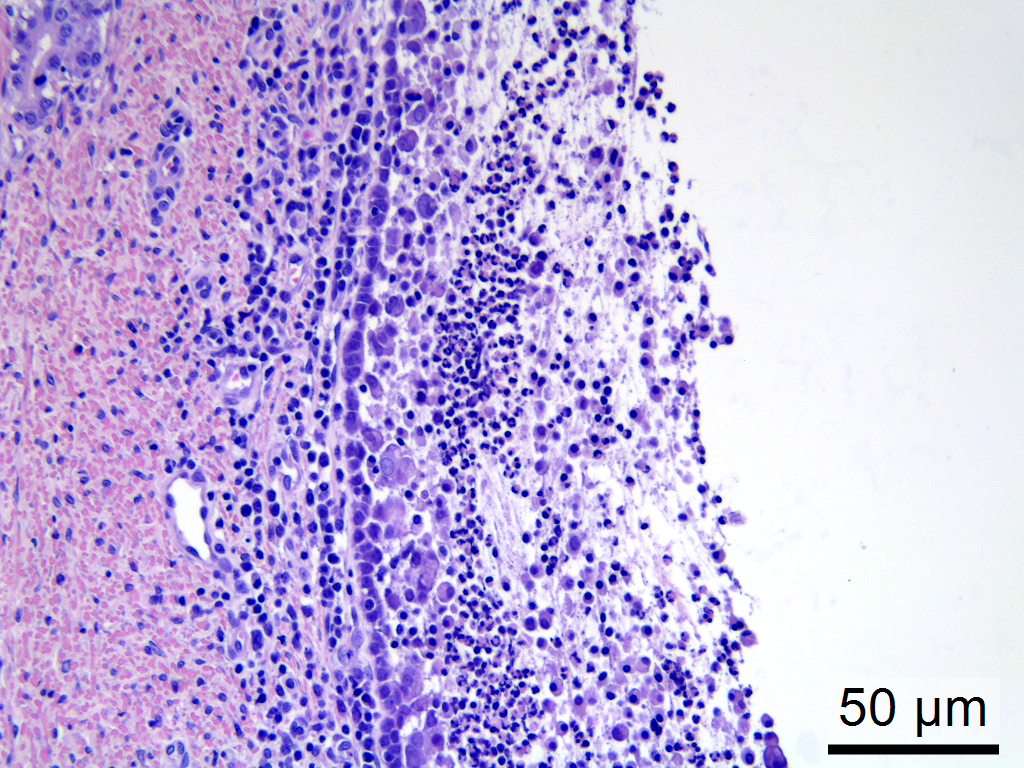

Supplement: S1 Fig — The histopathological changes observed were suppurative exudation, with disseminated epithelial erosion leaving only the basal cell layer and with infiltration of mononuclear cells in lamina propria. (TIF) [file ppat.1013405.s003.tif]

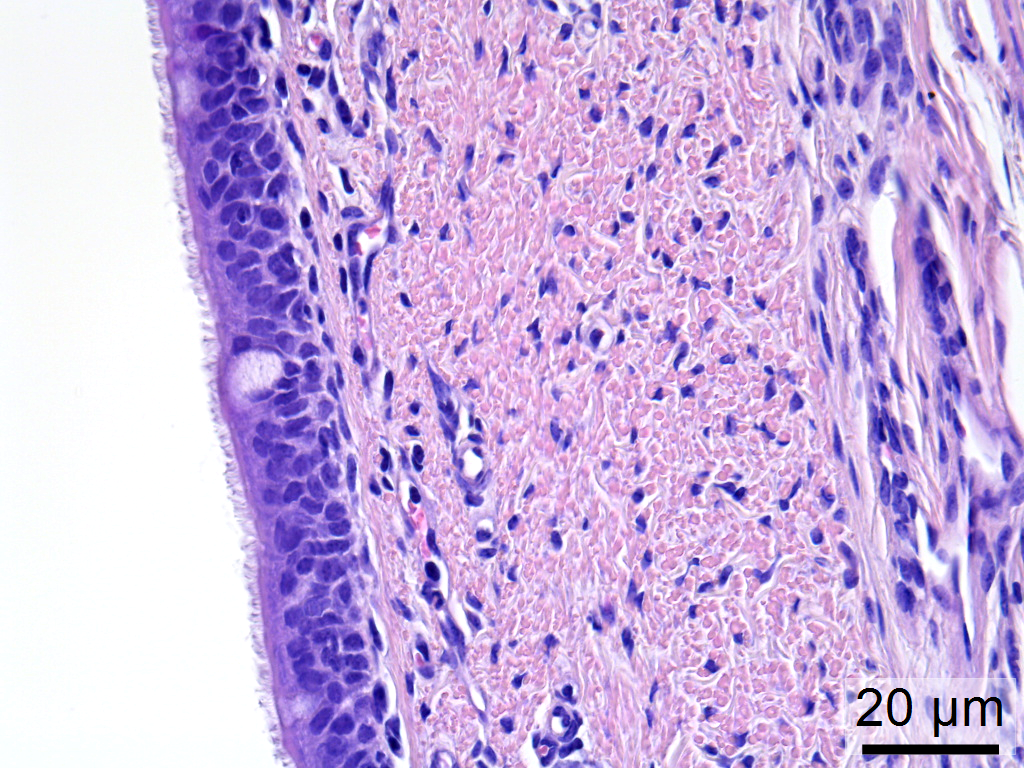

Supplement: S2 Fig — (TIF) [file ppat.1013405.s004.tif]
