# Supplementary material for: Experimental inoculation of pigs with porcine parainfluenza virus 1 revealed pathological manifestations in the upper respiratory tract
Source: PLoS Pathog. 2026 Mar 30;22(3):e1013405. doi: 10.1371/journal.ppat.1013405 (PMC13056255; doi:10.1371/journal.ppat.1013405)
Supplement: Attachment — Submitted filename: Reviewer_response_final_auresp_2.docx [file ppat.1013405.s010.docx]

**Additional Editor Comments:**

The abstract should be edited significantly to focus on the high level results and take home message of the study. As written, the abstract has too many experimental details (such as the n per group) that are best left for the main manuscript. The author summary is reasonable as written.

We agree. The abstract has been revised so the level of methodological details are lowered.

**Part II – Major Issues: Key Experiments Required for Acceptance**
Please use this section to detail the key new experiments or modifications of existing experiments that should be absolutely required to validate study conclusions.

Generally, there should be no more than 3 such required experiments or major modifications for a "Major Revision" recommendation. If more than 3 experiments are necessary to validate the study conclusions, then you are encouraged to recommend "Reject".

 We thank all Reviewers for taking their time to assess our manuscript and for the positive evaluation. We have considered all Reviewer’s suggestions and modified the manuscript accordingly. Our response to comments is highlighted in red.

Line number is referenced from the tracked changes version.

Reviewer #1: The authors mention on lines 346-348 and 467-469 that a pig in the control group had a positive ELISA result without detection of PPIV1 RNA, and they suggest that this represents a false positive. How specific is this serological assay and could a positive result be the product of cross-reactivity (suggesting an infection with a different, related antigen)? The authors state that the pigs were negative by ELISA on arrival, but is it possible that seroconversion for a different infection was delayed and appeared during the study?

The observation of a borderline positive results in a control pig by the whole-virus ELISA (wvELISA) warrants consideration, as it represents a potential limitation in the interpretation of study validity. Whole-virus ELISAs employing paramyxovirus antigens have previously been described as less specific than serological assays based on recombinant antigens (Mohan et al., 2006, *J. Virol. Methods*). Accordingly, a degree of non-specific or heterologous antibody binding cannot be fully excluded.

Nevertheless, under the controlled experimental conditions of the present study, environmental exposure of the control pigs to antigenically related viruses such as Newcastle disease virus, porcine rubulavirus, Sendai virus-like respiroviruses, or human parainfluenza viruses is considered highly unlikely. Moreover, the wvELISA employed has been validated against indirect immunofluorescence assay (IFA) and serum virus neutralization (SVN) in a large sample set, with a reported specificity of 99% at the selected cutoff (Welch et al., 2022, *BMC Veterinary Research*). On this basis, the occurrence of a false-positive result in control pigs sampled 14 days post-inoculation would be statistically unexpected, which could argue against a purely technical false-positive interpretation.

However, it should be noted that the observed S/P value (0.149) lies immediately above the cutoff (0.15) and within the range typically associated with analytical noise, borderline reactivity, or weak non-specific binding. When considered alongside the absence of compatible clinical signs, the lack of seroconversion in the second control pig, and consistently negative RT-PCR results throughout the study, a true PRV-1 specific antibody response appears unlikely.

Taken together, despite the theoretical possibility of assay cross-reactivity, the high assay specificity at this threshold, the lack of biological plausibility for exposure to antigenically related viruses in pigs, and the absence of virological evidence suggest that the finding is most consistent with non-specific ELISA reactivity rather than genuine PPIV-1 seroconversion.

We have included a sentence about possible cross-reactivity in the discussion lines: 501-507.

I suggest that the authors submit their raw alignments (lines 301-302) to ensure the repeatability of their genomic analyses.

We consider that as a reasonable request from other researchers and the alignment will be submitted in a “.fasta”-file format as a supplementary material (lines 330-331).

I strongly suggest that the authors include a table summarizing the results and timeline of the PCR, serological, and isolation detection attempts for all pigs enrolled in the study; this would make the results easier to follow.

We agree that this increases the readability of the manuscript and have included this as Table 1. All PCR-values are presented in Fig 2 and raw data in Supplementary file S1.

Reviewer #2: (No Response)

Reviewer #3: 1. Some of the information in the study design section of materials and methods would be more helpful in results section for readability. Figure 1 is also not referenced in the results section which I believe is a requirement of the journal. Moving some additional information into results would help with the requirement to reference figure 1 in the text.

We have included Figure 1 in the Results section (line 348), a more detailed description of the use of pooled nasal swabs as SOV inoculum, and the reason for choosing some of the SOV pigs as PPIV1 recipients (lines 344-348).
a. Line 313 could use some detail/clarification and additional information on attempts to rescue SOV and infect animals would be helpful.

We agree with this point and have included some additional information on the isolation attempts in cells (lines 129-144).

**Part III – Minor Issues: Editorial and Data Presentation Modifications**
Please use this section for editorial suggestions as well as relatively minor modifications of existing data that would enhance clarity.

Reviewer #1: Lines 59-66: In the introduction, consider including some background context on Nipah virus, another paramyxovirus that was identified after a large outbreak on a pig farm with devastating economic consequences for the industry (and onward zoonotic transmission to humans). This will help motivate the focus of this study.

We thank the reviewer for this suggestion. While Nipah virus is an important paramyxovirus, we have chosen to keep the introduction focused on viruses directly relevant to the scope of this study. Because Nipah virus differs substantially in its epidemiology and host context, we believe that including this background would broaden the introduction beyond its intended focus.

Relatedly, has the zoonotic potential of PPIV1/SOV been studied? Would be worth mentioning whether this has been assessed.
I would also consider mentioning the possibility of coinfection and PPIV1 infection facilitating other pathogens; this is explored in the discussion but would help motivate the focus of the study (and testing for multiple pathogens) if included in the introduction.

While the zoonotic potential of these viruses is an interesting topic, to our knowledge there is currently no evidence supporting zoonotic transmission, nor have studies specifically addressed this question. We therefore chose to focus this manuscript strictly on porcine viruses. Additionally, to avoid confusing the reader regarding the aims of the study, and given the limited existing knowledge about these viruses, we have chosen not to speculate on the potential consequences of coinfections in the introduction, but we will keep the porcine respiratory disease complex (PRDC) in the discussion.

Lines 77-78: more context about phylogenetic variation (or lack thereof) among PPIV1 strains would be helpful here.

We agree that this information is relevant to introduce the lack of phylogenetic variation and we have included this in the aim of the study (lines 103-106).

Line 118: where was the field study from which these SOV positive samples were collected?

We also agree on this point and the information has been included now (lines 129-131 and 142-144).

Line 139 and table S1: I would use the word sex instead of gender.

The word gender has been replaced with sex.

In figure 1, why are the SOV-inoculated pigs two different colours and the PPIV1-inoculated pigs one? Is this to indicate that half became recipients?

We agree that this was not clearly explained. More information has been added to the Figure legend.

The last two paragraphs of the discussion as well as the conclusion should be proofread for grammar and spelling.

These paragraphs have been carefully read and corrected for grammar and spelling.

The SOV results are discussed only briefly in the discussion and not at all in the conclusion. While inoculation was unsuccessful, I think this is a point worth highlighting and discussing the implications of, as it could indicate that this virus is less effective at producing infections in pigs.

We have included some more information regarding the difficulties in isolating SOV and suggested other cell lines to increase the success rate. Additionally, we have discussed the possibility of this virus being less effective at producing infections in pigs (lines 534-549).

Reviewer #2: Title and abstract
- Line 19 According to the ICTV, Porcine respirovirus type 1 (PPIV1) has been renamed Respirovirus suis. The authors are kindly requested to update the title and the entire text accordingly, and to include the appropriate ICTV reference and release version.

It is correct that the species name was renamed to *Respirovirus suis* from *Porcine respirovirus type 1*, but is the virus name remains porcine parainfluenza virus 1 (PPIV). The manuscript has been updated accordingly.

Introduction
- Lines 87-90 In reference no. 5 (ICTV website), the date of consultation is missing. KP Moreover, in the Orthopneumovirus genus, the muris species should be listed together with HRSV and BRSV, as indicated in the current ICTV taxonomy. Please update the Introduction accordingly.

The reference has been updated, and *Orthopneumovirus muris* (PVM) has been added to the mentioned orthopneumoviruses (lines 99-100).

Materials and methods
- Line 112 Could the authors please clarify whether the inoculum was removed after the one-hour incubation period?

The methodology has been clarified in lines 124-125.

- Lines 115-116 The authors are kindly requested to provide the reference for the RT real-time PCR method employed to verify the presence of viral nucleic acid. Please specify whether PPIV1 produced a cytopathic effect in the LLC-MK2 cells.

We agree that this information is important, and a reference to the RT real-time PCR has been added together (lines 138-139) and the information that no cytopathic effect was observed in the LLC-MK2 cells is mentioned in the results (lines 343-344).
- Lines 117-124 Please clarify whether both cell lines, LLC-MK2 and Calu-3, were used in the attempt to isolate SOV, as this point is not clearly stated in the manuscript. In addition, please specify the cycle threshold (Ct) values observed as positive in the RT-qPCR, and indicate which RT-qPCR assay was used by providing the appropriate reference. Since these methods are mentioned here for the first time, the corresponding references should be provided.

Thank you for mentioning this. The missing information is provided in the revised manuscript.

- Line 136 Please provide the reference for the ELISA test used to detect anti-PPIV1 antibodies.

A reference has been added at the end of this sentence (Reference 25).

- Line 142 The authors are kindly requested to specify which active substance was used for the anesthesia of the pigs.

We agree that this information is important; therefore, the use of active substances has been added.

- Figure 1 The depiction of the nasal swab at day 3 post-infection is missing and should be included.

Thank you for mentioning this. The day 3 post infection sampling was mistakenly included in the text and has been removed from the main text and the Figure legend.

- We suggest inserting Table S1 directly into the main text, as this would make the identification of the animals used in the experiment much clearer.

We agree that we use Animal ID numbers several times throughout the text and therefore have included the group and animal ID numbers in Table 1, placed in the main text together with summary findings. We have kept the weight and sex information as summary information (Table S1).

Reviewer #3: 1. You compare European and North America PPIV1 strains in line 70. It was unclear to me as a reader that we did not know how divergent these strains are (this is explored in Fig 8. Rewriting this to highlight that you will investigate this would be helpful. KP

We agree. The phylogenetic variation is not described and we have included this as an aim of the study (lines 103-106).
2. In paragraph line 85 you note that SOV was discovered recently, is there a year for this?

Yes, thank you for this suggestion, we have included the year in line 95.

3. Line 87 “SOV, which did not yet receive an official species designation” - should read “which has no yet received an official species designation”

Thank you for mentioning this; it has been corrected in the revised manuscript.
4. Line 497- should be “chances” instead of “changes" CK

Thank you for mentioning this, it has been corrected in the revised manuscript.
